# Nanoporous Anodic Alumina 3D FDTD Modelling for a Broad Range of Inter-pore Distances

**DOI:** 10.1186/s11671-016-1575-6

**Published:** 2016-08-12

**Authors:** Francesc Bertó-Roselló, Elisabet Xifré-Pérez, Josep Ferré-Borrull, Josep Pallarès, Lluis F. Marsal

**Affiliations:** Department of Electronic, Electric and Automatic Engineering, Universitat Rovira i Virgili, Avda. Països Catalans 26, 43007 Tarragona, Spain

**Keywords:** Nanoporous anodic alumina, Numerical modelling, FDTD simulation, Interface texturization, Anion layer

## Abstract

The capability of the finite difference time domain (FDTD) method for the numerical modelling of the optical properties of nanoporous anodic alumina (NAA) in a broad range of inter-pore distances is evaluated. FDTD permits taking into account in the same numerical framework all the structural features of NAA, such as the texturization of the interfaces or the incorporation of electrolyte anions in the aluminium oxide host. The evaluation is carried out by comparing reflectance measurements from two samples with two very different inter-pore distances with the simulation results. Results show that considering the texturization is crucial to obtain good agreement with the measurements. On the other hand, including the anionic layer in the model leads to a second-order contribution to the reflectance spectrum.

**Electronic supplementary material:**

The online version of this article (doi:10.1186/s11671-016-1575-6) contains supplementary material, which is available to authorized users.

## Background

In the last years, nanoporous materials have gained great relevance in fields such as biotechnology [1–3], medical sciences [4–8] and energy [9, 10]. Optical properties of such materials are of particular interest as many of their applications are based on their interaction with photons, as for instance in optical biosensing or in photovoltaic energy conversion [11–16]. Thus, the ability to predict the optical behaviour of porous materials is crucial to design and optimize the material structure with a view to its applications. Therefore, in this sense, the numerical modelling of the optical properties of the materials provides a theoretical framework for the analysis of their optical behaviour and of the performance of the devices based on them. For instance, in optical biosensing based on porous materials, a platform to conduct a selective identification of the analytes is required [17]. In this line, nanoporous materials have experienced a boom as sensing platforms to be used in biosensing devices. These materials can act like a receptacle of physical objects, being more suitable in comparison of non-porous materials because of their properties of high sensitivity and selectivity [18]. Furthermore, a biosensor requires also a transduction mechanism by which the detection of the analytes becomes a measurable and quantifiable signal. For this purpose, there are some methods of sensing: Kumeria et al., in ref. [19], show a classification in optical and physical detection methods with a detailed description of each type. The high surface-to-volume ratio property of the nanoporous materials makes them particularly suitable as a platform for sensing in optical biosensors. This aspect enhances the optical signal corresponding to the interaction of light with the structure and with the analytes.

The production of nanoporous materials can be done from electrochemical techniques such as porous silicon (pSi) and nanoporous anodic alumina (NAA). In particular, and because of their physical and chemical properties, NAA is a good platform for use in the development of devices and applications such as those related to sensing, drug delivery and energy. But it is especially suitable for use in optical biosensing for their optical properties in the visible range [20, 21], stability under a wide range of biological conditions [19] and great tunability of geometry and structure [22, 23]. The optical modelling of NAA has to take into account its different geometrical and chemical characteristics. NAA is a self-ordered porous material with cylindrical pores perpendicular to the surface in a hexagonal arrangement, produced by electrochemical etching of the aluminium. The main geometrical parameters to be considered when modelling the optical properties are the inter-pore distance (*d*_int_), the pore diameter (*d*_p_), the barrier layer thickness (*t*_b_) and the pore length (*L*), in addition to the optical constants of the different material constituents. The mentioned geometrical parameters are very sensitive to the anodization conditions like the anodization voltage, the electrolyte type, pH and temperature. The acid electrolytes usually used are sulphuric, oxalic and phosphoric acids, given different structural parameters for each one [19].

In addition to these geometrical parameters, the numerical modelling of NAA has to take into account other geometrical or structural features, such as the fact that the interfaces in the material are not perfectly flat or that the oxide is not homogeneous. During the electrochemical process of the alumina formation, a texturization of the interfaces occurs: after the first step in the usual two-step anodization procedure [22], the obtained porous oxide is removed to obtain a texturized aluminium which provides a self-ordered array of hemispherical concavities in the metal which act as nucleation sites for the pores in the second step. Such hemispherical concavities remain in the metal-barrier layer as the porous oxide grows in the second step. Additionally, anions are incorporated from the electrolyte acid to the NAA influencing the distribution of the chemical composition of the oxide and thus the spatial distribution of their optical properties. The quantity of anions and their distribution into the oxide depends on the anodization conditions, particularly on the type of the acid electrolyte [24]. More precisely, NAA structures exhibit a layered structure in the chemical composition of the pore wall oxide, being the nearest layer to the pore and the one with a higher anion concentration. The number of layers that forms this layered structure differs for different studies: Kumeria et al. [25] reported that the incorporated anions are distributed to four layers in an onion-like manner. On the other hand, Thompson et al. [26] reported that the chemical composition shows a dual layer structure. In any case, these anions have properties of both absorption and emission of light. The existence of absorption is because of the oxygen vacancies and the incorporation of anions [27, 28]. Thus, the incorporation of the anionic species influences the optical constants of the oxide, consisting of a deviation from the refractive index and extinction coefficient of bulk alumina.

As we mentioned above, it is important to predict the optical behaviour of NAA. The transfer-matrix method (TMM) is a widespread method for modelling the optical behaviour of micro- or nanostructured materials such as thin films [29] or photonic crystals [30, 31]. In the case of thin film modelling, it considers flat interfaces and homogeneous materials. Usually, the effective medium approximation (EMA) is used in TMM to obtain the effective refractive index when one of the materials in the structure is actually a mixture of materials [32]. Several effective medium approximations exist, such as that of Bruggeman or Maxwell-Garnett [33, 34]. Thus, when modelling NAA with the TMM, EMA can be used to obtain the effective refractive index of the porous oxide layer. However, in NAA, the interfaces are not flat, and in some cases (such as the NAA produced with phosphoric acid electrolyte), EMA is not a good approximation as the dimensions (pore diameter or inter-pore distance) of the different constituents of the media have values between 350 and 500 nm, which are in the order of the wavelength of light if applications in the visible range are desired. Consequently, although TMM and EMA can be adequate for some cases of NAA, they are clearly not adequate for all the range of structures. Alternative numerical modelling methods exist: finite difference time domain (FDTD) [35, 36], boundary element method (BEM) [37, 38], rigorous couple-wave analysis (RCWA) and others. In this work, we have chosen FDTD because of its accuracy and ability to deal with complex structures. The objective of this work was to evaluate the capabilities of FDTD as a framework for the numerical modelling of the optical properties of NAA, which is able to take into account all the features described above: broad range of dimensions, texturization of the interfaces and non-homogeneity of the oxide. Furthermore, we also establish the conditions in which EMA-TMM is adequate for the simulation of NAA by comparing its results to FDTD.

To achieve this, we measured reflectance spectra of real NAA samples fabricated with oxalic acid electrolyte (short inter-pore distance) and with phosphoric acid electrolyte (long inter-pore distance) and we compared the experimental results with the simulation results using the different numerical methods and considering different models for the geometrical and optical properties of the NAA structures.

## Methods

### NAA Fabrication

The NAA samples were fabricated following the well-known two-step anodization process [39, 40]. The first step is carried out in a solution of an electrolytic acid and under potentiostatic conditions. The pores nucleate on the aluminium substrate and the oxide starts growing perpendicularly to the substrate surface. Oxide growth starts with a disordered porous layer until by the barrier layer action the pores become self-ordered in a hexagonal lattice. Next, the porous oxide is removed leaving the aluminium substrate texturized with a hexagonal array of hemispherical concavities. The second anodization step is performed under the same conditions as the first one. In this process, the pores form at the centre of the concavities and the oxide layer grows in a hexagonal arrangement, although the ordering is not perfect but broken into domains.

Two kinds of NAA samples with different structural parameters were obtained. Additional file 1: Figure S1 shows top-view SEM images of both kinds of NAA samples. The inter-pore distance (*d*_int_) and the pore diameter (*d*_p_) are estimated from these SEM images. In the first case, we used oxalic acid (0.3 M, 40 V, 5 °C) as the electrolyte acid. The major structural parameters of the samples obtained with this acid were as follows: *d*_int_ = 100 nm, *d*_p_ = 21.5 nm and pore length (or equivalently, porous layer thickness) *L* = 1200 nm. The second sample was obtained using phosphoric acid (1 % wt., 174 V, 0 °C) as the electrolyte. In this case, the structural parameters characterizing the sample were as follows: *d*_int_ = 440 nm, *d*_p_ = 125 nm and *L* = 2400 nm. Both kinds of sample maintain the aluminium substrate.

### NAA Characterization

The reflectance spectra of the samples were measured using a Lambda 950 spectrophotometer from PerkinElmer (Whaltham, MA, USA) equipped with a tungsten lamp as the light source and using a universal reflectance attachment. The measurements were done in the 400−2000-nm range at quasi-normal incidence (6°). Additional file 1: Figure S2 summarizes both spectra measurements. It shows the measured reflectance spectra for both kinds of samples: one for short inter-pore distances, that is *d*_int_ = 100 nm, and another one for long inter-pore distances, *d*_int_ = 440 nm. Both measured spectra show an oscillating behaviour as a result of the Fabry-Pérot interferences in the NAA thin film. Nevertheless, the spectrum for *d*_int_ = 440 nm shows a strong reduction of reflectance in the visible range.

### EMA-TMM Calculations

For the EMA-TMM calculations, the alumina porous layer can be described by a mixture of air and the host alumina. Thus the effective refractive index of this layer would be a combination of the refractive indices of air and alumina, provided by the effective medium approximation. EMA permits also to take into account the substrate texturization, up to some extent, as a very thin film composed of mixture of aluminium and oxide. TMM allows us to consider the structure as a multi-layered one with homogeneous layers. The effective refractive index corresponding to the porous layer was calculated using Bruggeman’s formula, with the refractive index of the aluminium taken from ref. [41]. The thickness and the porosity of the porous layer were taken from the experimental data described previously: *L* = 1200 nm for short inter-pore distance NAA with a porosity of 4.19 % and *L* = 2400 nm for long inter-pore distance NAA with a porosity of 7.31 %. The EMA-TMM simulations were designed and performed with a computational tool developed by our group.

### FDTD Calculations

Additional file 1: Figure S3 shows the computational domain used in the simulations, with dimensions of *d*_int_ nm in the *X* direction, $\sqrt{3}$·*d*_int_ nm in the *Y* direction and 14,000 nm in the *Z* direction, for each inter-pore distance *d*_int_ considered. The NAA structure corresponds to a unit cell of a hexagonal lattice distribution of air pores in an aluminium oxide matrix onto an aluminium substrate and is placed in the centre of the computational domain. The optical constants of the materials (refractive index and extinction coefficient) were taken from the material database within the software, based in ref. [41]. Symmetry in the *X* and *Y* directions is applied to reduce the computational cost, and periodic boundary conditions in these directions were applied to account for an infinite structure. In the *Z* direction, to avoid unwanted reflections, we considered perfectly matched layers (PMLs) as a boundary condition. The source was a plane wave incident on the porous oxide layer side with wavelengths in a range from 400 to 2000 nm, propagating backwards in the *Z* direction and perpendicular to the structure (*X*-*Y* plane). Finally, to collect the reflectance as a function of wavelength, a data monitor that covers the whole computational domain is placed at the top of the structure (Additional file 1: Figure S3a).

**Fig. 1:**
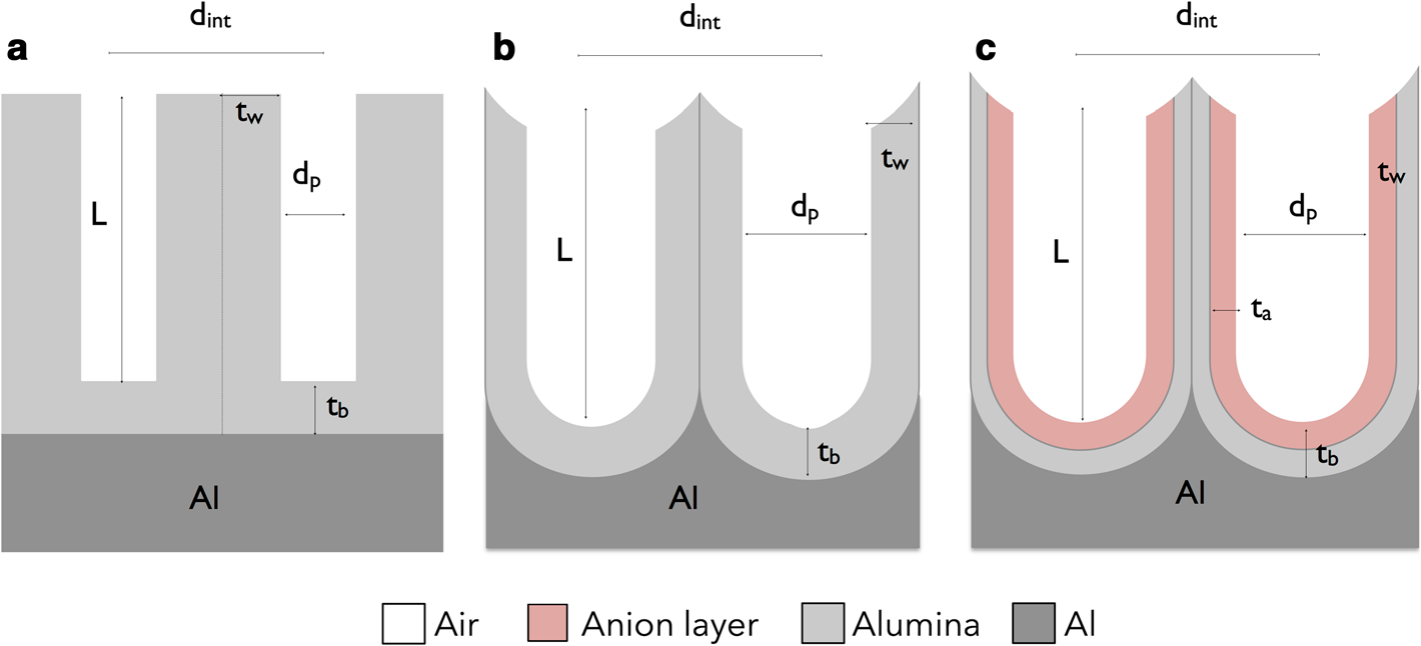
Schematic cross sections of the different geometrical models. **a** First model with flat interfaces between the aluminium substrate and the porous layer. **b** Second model with the texturization of the interfaces with hemispherical concavities. **c** Third and fourth models with the dual layer structure in the pore walls, without and with absorption, respectively

Additional file 1: Figure S3b shows a planar view of the hexagonal unit cell designed to reproduce the hexagonal arrangement of cylindrical pores of the NAA. Four different models for this cell are taken into consideration. The models differ by the increasing complexity of the geometry and of the refractive index distribution. Figure 1 shows a schematic picture of these models. In the first model, we considered a layer of NAA with cylindrical pores on a flat aluminium substrate. The top surface and the alumina barrier layer were also flat. Figure 1a shows a schematic cross section of the model used in this first stage, where *d*_int_ is the inter-pore distance, *d*_p_ the pore diameter, *t*_w_ the pore wall thickness, *L* is the pore length and *t*_b_ is the barrier layer thickness. Additional file 1: Table S1 summarizes the values of these NAA structural parameters, *d*_p_ = 21.5 nm and *L* = 1200 nm for *d*_int_ = 100 nm, and *d*_p_ = 125 nm and *L* = 2400 nm for *d*_int_ = 440 nm, used for the simulations. Porosity of each structure is determined from the structural parameters. In both cases, the width of the substrate was taken as 4000 nm.

Next, with the second model, the texturization of the alumina barrier layer was introduced. With this, the interface between the aluminium substrate and the oxide becomes texturized with hemispherical concavities. Simultaneously, an equivalent texturization of the alumina barrier layer and the top surface of the alumina layer was also incorporated. Figure 1b shows a schematic cross section of the improved pore morphology with texturized interfaces. The structural parameters are the same as those depicted in Fig. 1a.

In the third model, Fig. 1c, we take into account the anion incorporation by introducing the double layer structure in the pore wall, in addition to the texturized interfaces considered in the second model. We modelled cylindrical shells of a dielectric material with a refractive index different from that of the bulk aluminium oxide. The outer shell, from now on designated as the *anionic layer*, extends from the aluminium oxide-air interface within the pore to about two thirds of the barrier layer for structures created from phosphoric acid [26]. Additional file 1: Table S1 also shows the refractive indexes and the width of the anionic layer we considered. Figure 1c shows a schematic cross section of the texturized model with the double layer structure in the pore wall. The anionic layer is depicted in a different shade in Fig. 1c, and *t*_a_ indicates its width. Additional file 1: Table S1 also shows the considered refractive indexes and anionic layer widths. Finally, in the fourth model, we take into consideration the fact that these anions have properties of both absorption and emission of light. Therefore, a complex refractive index for the anionic layer is used in the simulations, as reported in Table S1.

### Procedure to Take into Account the Non-periodicity of the Pores

The FDTD simulations described above provide the numerical modelling of a NAA structure which is completely periodic, with a perfectly hexagonal arrangement and with absolutely cylindrical pores. The simulation of a perfect periodic structure results in reflectance spectra with peaks related to diffraction modes, especially for inter-pore distances close to the wavelength range. However, it is known that pore ordering in NAA is not perfect as the order is broken into domains. As a consequence, the measured reflectance spectra do not show any diffraction-related features. Furthermore, the real samples show a dispersion in all of their characteristic geometrical parameters (inter-pore distance, pore diameter and pore length).

This breaking of the periodicity and the dispersion in the geometrical parameters is taken into account in the FDTD simulations, for the case of the long inter-pore distance, by calculating reflectance spectra for a set of unit cells with geometrical characteristics in a range close to the nominal values reported above and obtaining their average. The considered range for the inter-pore distance was from 430 to 450 nm in steps of 10 nm; for the pore diameter, was from 120 to 130 nm each 5 nm; and for the pore, was from 2360 to 2440 nm with intervals of 20 nm.

## Results and Discussion

### Limits of the EMA-TMM Approach

**Fig. 2:**
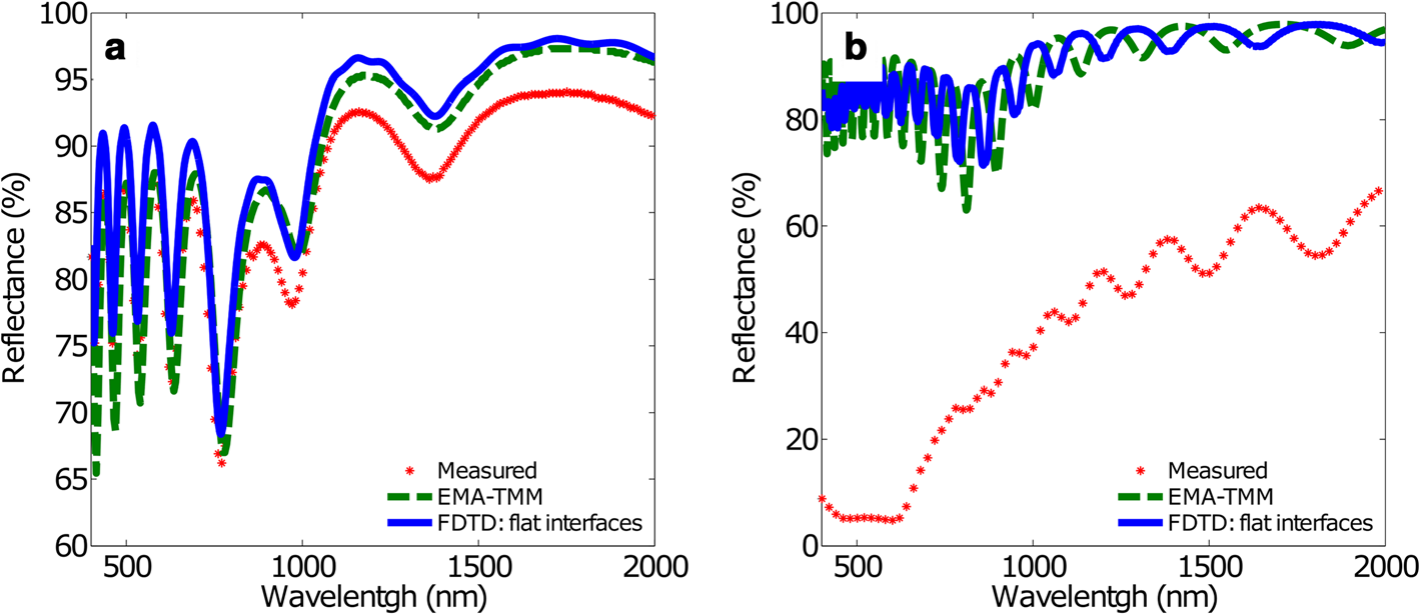
Measured and calculated reflectance spectra using EMA-TMM and FDTD with the flat interfaces model. **a** *d*_int_ = 100 nm and **b** *d*_int_ = 440 nm. The *red dots* represent the measured reflectance spectra, the *green dashed line* the EMA-TMM results and the *blue solid line* the FDTD result

Figure 2 compares the reflectance spectra obtained using the usual EMA-TMM approach (dashed line) with those of the FDTD method considering flat interfaces (first method, solid line) and with the experimental measurements on actual real samples (dots). Figure 2a corresponds to NAA with a short inter-pore distance (*d*_int_ = 100 nm) while Fig. 2b corresponds to the long inter-pore distance (*d*_int_ = 440 nm). The reflectance scales of these two figures are adjusted to maximize visibility of the results.

The measured spectra for short inter-pore distances show an oscillating behaviour as a result of the Fabry-Pérot interferences in the NAA thin film. Such behaviour can also be observed in the spectrum for long inter-pore distances in the IR range (between 900 and 2000 nm). However, in this latter case, the reflectance shows a strong reduction in the visible range. Both simulation methods are able to reproduce the oscillation behaviour, but fail to predict the decrease in reflectance for long inter-pore distance. Furthermore, a closer analysis reveals that for short inter-pore distance, a minor but noticeable discrepancy in the amplitude of the oscillations appears in the visible range, in particular between the measured reflectance and the EMA-TMM values.

These discrepancies are due to two factors: first, the EMA assumes that the different materials integrating the effective medium have characteristic sizes much smaller than the wavelength. Such assumption is not fulfilled as the wavelength approaches the visible range, especially for the long inter-pore distance structures. Second, although the FDTD method is able to take into account naturally the different characteristic sizes of the structures, both methods fail to predict the dramatic reduction in reflectance for long inter-pore distance in the visible region, what leads to the conclusion that the assumption of flat interfaces is not adequate.

### Patterning of the Aluminium Substrate

**Fig. 3:**
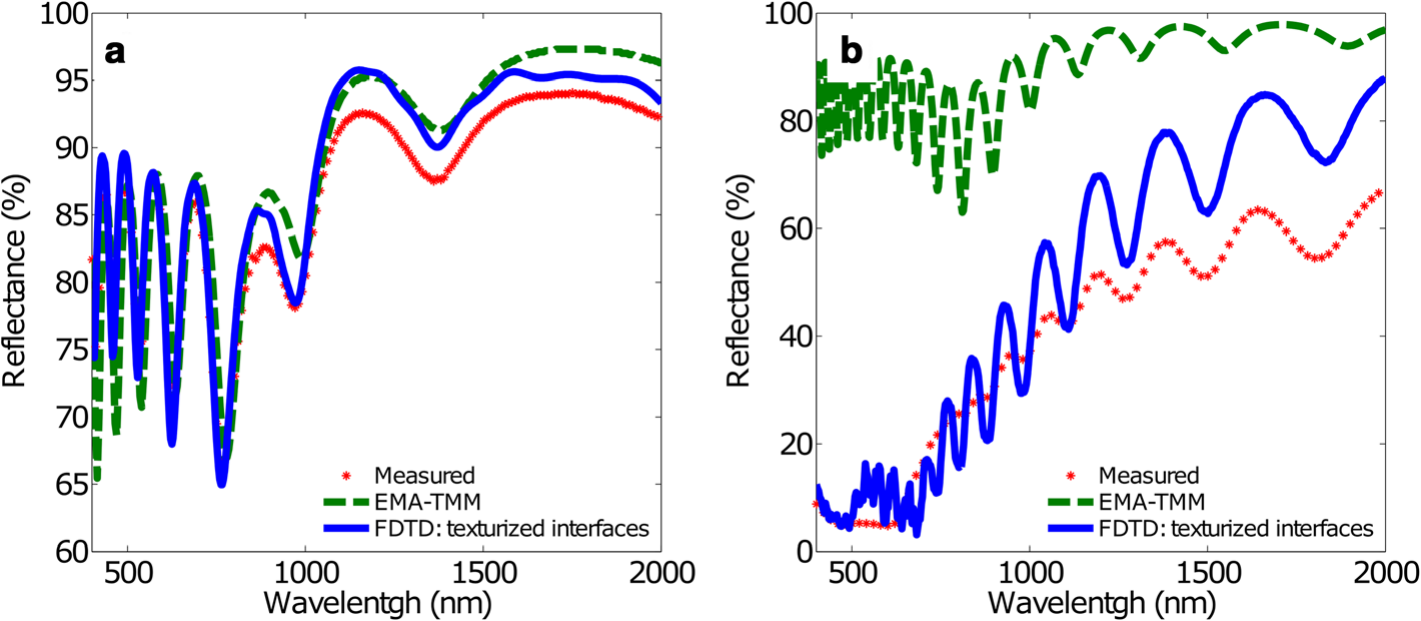
Measured and calculated reflectance spectra using EMA-TMM and FDTD with the texturized interface model. **a** *d*_int_ = 100 nm and **b** *d*_int_ = 440 nm. The *red dots* represent the measured reflectance spectra, the *green dashed line* the EMA-TMM results and the *blue solid line* the FDTD result

Thus, the two conclusions we can draw from Fig. 2 lead us to consider the texturing of the interfaces, what renders FDTD as the most suitable method for the simulations. Figure 3 shows the same measured spectra and the same calculated spectra with EMA-TMM as in Fig. 2. In addition, Fig. 3 also shows the result of FDTD considering the texturized interfaces of the second model. EMA-TMM spectrum is included as a reference to assess the changes introduced by texturing. Thus, for short inter-pore distances (Fig. 3a), the agreement between calculated and measured spectra is better than in the case of FDTD without texturing the metal-oxide interface: in the IR region, the differences are smaller, while in the VIS region the amplitude of the oscillations is much better adjusted.

Instead, for long inter-pore distance structures (Fig. 3), a crucial change in the calculated optical behaviour is observed: FDTD is able to reproduce the strong reduction in the reflectance in the VIS range, but still cannot adjust with precision the oscillations. This dramatic reduction in the reflectance may come from the scattering produced by the interaction of light with the nanostructured NAA. When the characteristic dimensions of the structure approach the wavelength of light, this scattering is stronger and becomes essential when we also consider the metal-oxide interface texturization. Thus, we can conclude that for an appropriate modelling of NAA structures in a wide range of inter-pore distances, it is crucial to take into account the texturization of the metal-oxide interface.

### Incorporation of the Anion Layer

**Fig. 4:**
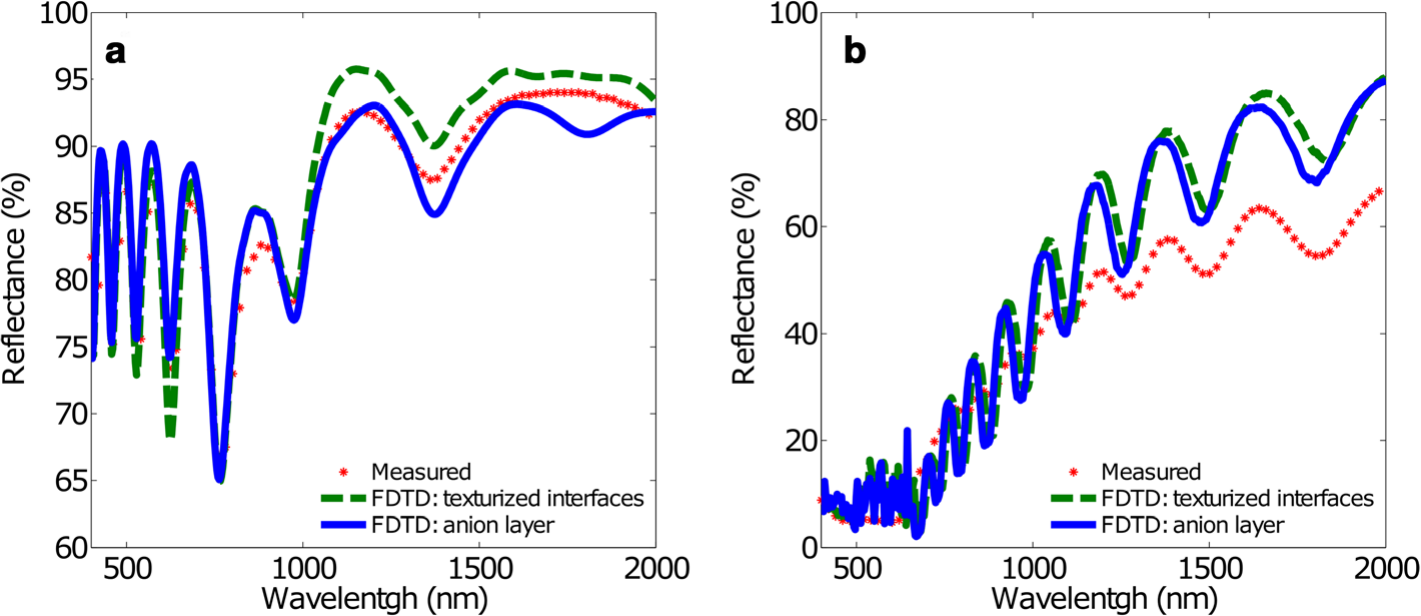
Measured and calculated reflectance spectra using FDTD with the texturized interface model and the anionic layer model. **a** *d*_int_ = 100 nm and **b** *d*_int_ = 440 nm. The *red dots* represent the measured reflectance spectra, the *green dashed line* the FDTD result for the texturized interface model and *the blue solid line* represents the FDTD result for the anionic layer model without absorption

As we have shown above, interface texturization leads us to a better description of the optical behaviour of the NAA structures. This improvement can be observed both for short and for long inter-pore distance NAA structures. However, some discrepancies with respect to the experimental data can still be found. For this reason, the well-known fact that the pore walls have a double layer structure has been taken into consideration in the third model. The calculated spectra considering this double layer structure are shown in Fig. 4. As a reference, the measured spectra depicted in the previous figures together with the calculated spectra shown in Fig. 3 are also included, in order to evaluate the contribution to the spectra of each incremental step in model complexity.

Introducing the double layer structure in the simulations leads to a small adjustment of the calculated spectrum. In Fig. 4a, corresponding to the short inter-pore distance, the variation is very small in the VIS region, while in the IR region a reduction of the reflectance is obtained, leading to a better agreement with the experimental data. This reduction in reflectance in the IR region is also observed for long inter-pore distance in Fig. 4b, together with a reduction in the amplitude of the oscillations. Thus, we can conclude that the addition of this double layer structure into the model improves noticeably the simulation of the optical properties of the NAA.

### Absorption in the Dual Layer Pore Wall Structure

It is known that the structure of the double layer of the pore wall oxide is due to the incorporation of anions from the electrolyte to the NAA. These anions have properties of both absorption and emission of light. In the fourth model, an absorptive term has been introduced to the anionic layer of the double layer structure. The addition of the absorptive term in this layer entails a change in the real and imaginary component of the refractive index of the concerned area.

**Fig. 5:**
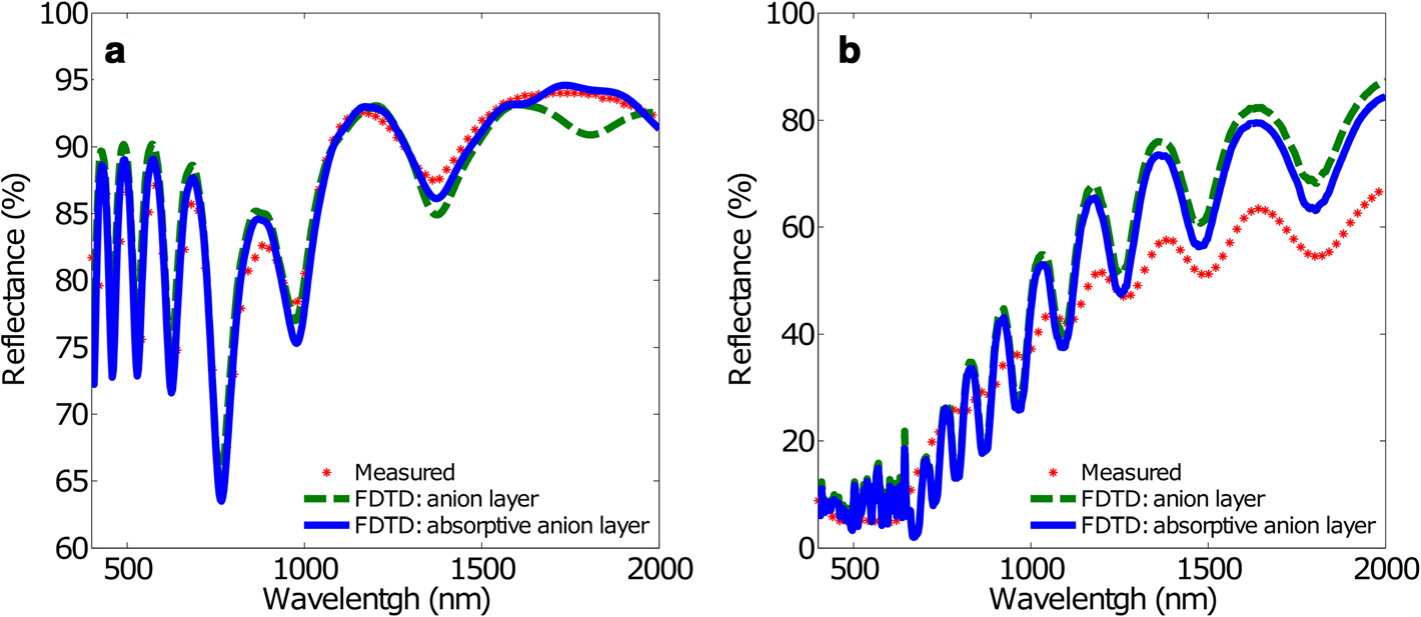
Measured and calculated reflectance spectra using FDTD with the anionic layer model, with and without absorption. **a** *d*_int_ = 100 nm and **b** *d*_int_ = 440 nm. The *red dots* represent the measured reflectance spectra, the *green dashed line* the FDTD result for the anion layer model without absorption and the *blue solid line* the FDTD result for the anionic layer model with absorption

Figure 5 summarizes the obtained simulation results considering absorption in the anionic layer. As a reference, the measured reflectance and the calculated FDTD spectra considering the double layer structure without absorption in their pore wall oxide are shown. In the plot, the solid line corresponds to a zero absorption coefficient while the dashed line to nonzero absorption. Figure 5a shows a slight reduction of the amplitude of the oscillations in reflectance in the VIS region for the anionic layer with absorption with respect to the corresponding anionic layer without absorption. In the IR region, the adjustment considering absorption is even better because it eliminates a strange oscillation that the experimental measurement does not show. Figure 5b shows a shift of the reflectance spectrum of the anionic layer with absorption towards shorter wavelengths relative to that obtained with the corresponding anion layer without absorption. In addition, Fig. 5b shows a slight reduction of the average reflectance for the anionic layer with absorption. The consideration of an absorptive anionic layer into the double layer pore wall oxide causes a slight correction effect on the spectral Fabry-Pérot oscillations, which represents a fine adjustment of the spectrum.

## Conclusions

In this work, we presented a numerical procedure based on 3D FDTD simulations able to predict the optical behaviour of NAA for a broad range of inter-pore distances. Reflectance measurements were simulated for NAA obtained with oxalic (inter-pore distance smaller than wavelength) and with phosphoric (inter-pore distance of the order of wavelength) electrolytes.

Different geometrical models have been considered in the simulations: (i) a first model consisting of flat interfaces between the aluminium substrate and the porous layer, (ii) a second one in which the texturization of the interfaces with hemispherical concavities (produced by the fabrication method) is considered, (iii) a third model which introduces the known fact that the pore walls have a dual layer structure with different optical properties and (iv) a fourth model in which absorption is included in the optical constants for the outer shell of the dual layer.

The results obtained with the consideration of flat interfaces between the aluminium substrate and the porous layer allow us to establish the limits of the EMA-TMM approach and confirm FDTD as a more appropriate numerical method to employ. FDTD in comparison with EMA-TMM leads to the same result for short inter-pore distance, while there is a discrepancy in the VIS range for long inter-pore distance. On the other hand, both methods fail to predict the decrease in reflectance at the VIS range, making the assumption of the flat interfaces not pertinent. If the texturization of the interfaces is taken into account, the reduction of reflectance in the VIS for long inter-pore distance is obtained, while a good agreement for the short inter-pore distance is still observed. With a further refinement of the model, considering the dual structure of the pore walls, both without or with a certain amount of absorption, leads to a slight correction of the spectrum and to a better agreement with the experimental measurements.

With these results, we have shown that it is critical to consider the interface texturization in order to obtain accurate predictions of the optical behaviour of NAA, valid for a wide range of structure sizes. On the other hand, considering in the numerical models other features such as the dual structure of the pore walls only gives an incremental correction, although they permit a better agreement with actual measurements.

## Abbreviations

EMA, effective medium approximation; FDTD, finite difference time domain; NAA, nanoporous anodic alumina; PMLs, perfectly matched layers; TMM, transfer matrix method; VIS, visible

## Additional File

Additional file 1: (DOC 400 kb)
