# Supplementary material for: Nanoporous Anodic Alumina 3D FDTD Modelling for a Broad Range of Inter-pore Distances
Source: Nanoscale Res Lett. 2016 Aug 12;11:359. doi: 10.1186/s11671-016-1575-6 (PMC4987547; doi:10.1186/s11671-016-1575-6)
Supplement: Additional file 1: — (DOC 400 kb) [file 11671_2016_1575_MOESM1_ESM.doc]

Additional file 1

**3D-FDTD MODELLING OPTICAL BEHAVIOUR OF LONG INTERPORE DISTANCE NANOPOROUS ANODIC ALUMINA**

**
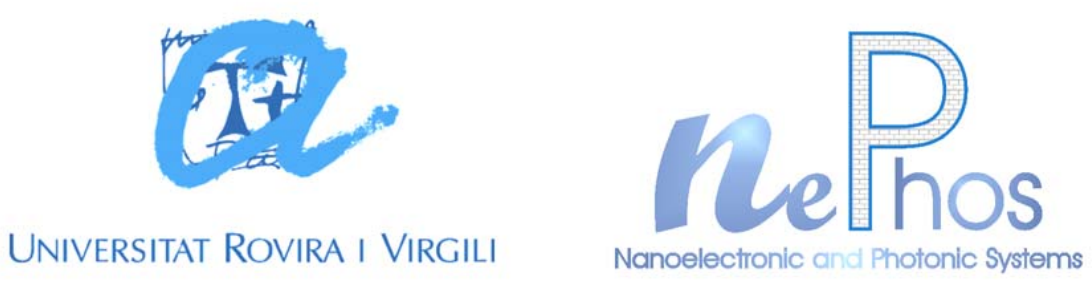
**

FRANCESC BERTÓ-ROSELLÓ, ELISABET XIFRÉ-PÉREZ, JOSEP FERRé-BORRULL, Josep Pallarès, LLUIS F. MaRSAL*

Nano-electronic and Photonic Systems (NePhoS)

Departament d’Enginyeria Electrònica, Elèctrica i Automàtica

Universitat Rovira i Virgili.

Avda. Països Catalans 26, 43007 Tarragona, Spain.

**Figure S1: Top SEM images of the NAA structures.** (a) Top view SEM image for short inter-pore distance NAA. (b) Top view SEM image for long inter-pore distance NAA.


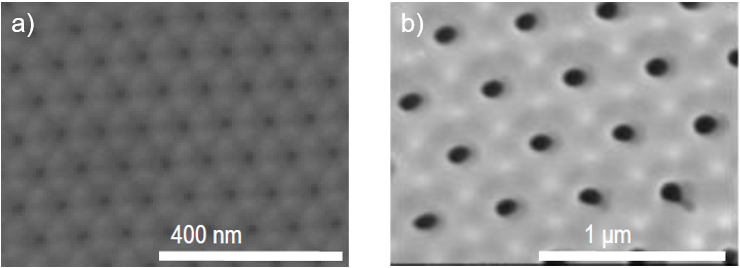


**Figure S2: Measured reflectance spectra of NAA samples.** Measured reflectance spectra of NAA samples with d_int_ = 100 nm (fabricated with oxalic acid electrolyte), and d_int_ = 440 nm (with phosphoric acid electrolyte).

**Figure S3: Schematic FDTD computational features.** (a) Schematic view of the computational domain: R denotes the reflectance monitor, NAA denotes the structure, PML denotes de perfect matched layers and the source are explicitly indicated. (b) Planar view of the FDTD unit cell: the black circles denote the pores in a hexagonal arrangement, while the red square denotes the unit cell in the XY plane.


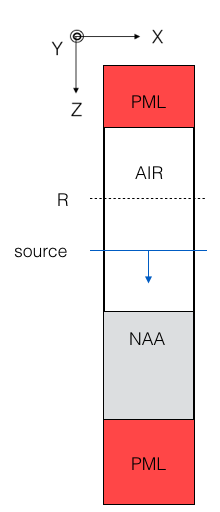
**a) b)**

**
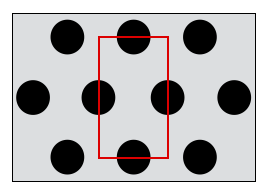
**

**Table S1. Structural parameters of the NAA for each inter-pore distance considered.**

| Structural parameter | d_int_ = 100 nm | d_int_ = 440 nm |
| --- | --- | --- |
| L | 1200 nm | 2400 nm |
| d_p_ | 21.5 nm | 125 nm |
| t_b_ | 0.5·d_int_ | 0.5·d_int_ |
| t_a_ | t_b_ · 2/3 | t_b_ · 2/3 |
| nanionic_layer | 1.67 | 1.67 |
| nabsorptive_anionic_layer | 1.67 + 0.002·i | 1.67 + 0.002·i |
